# The Perception of the Body Condition of Cats and Dogs by French Pet Owners and the Factors Influencing Underestimation

**DOI:** 10.3390/ani13233646

**Published:** 2023-11-25

**Authors:** Tiphaine Blanchard, Sara Hoummady, Damien Banuls, Mélanie Roche, Aurélie Bynens, Michel Meunier, Natalia Dos Santos, Emna Tissaoui, Pétra Rouch-Buck, Marco Fantinati, Nathalie Priymenko

**Affiliations:** 1 Ecole Nationale Vétérinaire de Toulouse ENVT, 31300 Toulouse, France; damien.banuls@envt.fr (D.B.); melanie.roche_16@envt.fr (M.R.); petra.rouchbuck@envt.fr (P.R.-B.); nathalie.priymenko@envt.fr (N.P.); 2 Ecole Nationale Vétérinaire d’Alfort, 94704 Maisons-Alfort, France; sara.hoummady@unilasalle.fr (S.H.); natalia.santos@vet-alfort.fr (N.D.S.); emna.tissaoui@vet-alfort.fr (E.T.); 3 Institut polytechnique Unilasalle Rouen, 76130 Mont-Saint-Aignan, France; 4 Fédération des Fabricants d’Aliments pour Chiens, Chats, Oiseaux et autres animaux familiers, FACCO, 75010 Paris, France; aurelie.bynens@facco.fr; 5 Hill’s Pet Nutrition France, 06560 Valbonne, Francemarco_fantinati@hillspet.com (M.F.); 6 Toxalim, Université de Toulouse, INRAE, ENVT, 31300 Toulouse, France

**Keywords:** canine, feline, obesity, overweight, owner, perception, veterinarian

## Abstract

**Simple Summary:**

Obesity is a significant health concern among companion animals, particularly dogs and cats, with numerous detrimental health implications. Many pet owners struggle with managing their pets’ weight, and they often underestimate their pets’ body condition. This study, held in France from 2020 to 2022, revealed that about one quarter of pet owners underestimate their pets’ body condition. Having children was linked to this underestimation for both dog and cat owners. This discovery highlights the importance of taking a comprehensive approach to the health and well-being of pets and emphasizes the need for a holistic “One Health” strategy.

**Abstract:**

Managing pet obesity relies heavily on the active involvement of owners; however, a key challenge arises from misperceptions about their own pet’s body condition. Given evolving societal dynamics like the body positivity movement, understanding owners’ perceptions is increasingly pivotal. To evaluate the differences in owners’ perception, this study compared the use of verbal and visual body condition score scales versus the established nine-point body condition score system. The factors linked to underestimation were further specifically investigated. Owners of healthy adult dogs and cats attending vaccination consultations in Veterinary Hospitals in France between 2020 and 2022 were recruited. They were required to assess their pets’ body condition initially using an oral description and then with the nine-point BCS visual scale. Their assessments were then compared with the BCS determined by veterinary health care personnel, considered the primary investigator. A total of 304 dogs and 270 cats were included in the study. It was observed that 27% of dog owners and 24% of cat owners underestimated their pets’ body condition. Among dog and cat owners, factors associated with the underestimation of body condition were the pets’ overweight status and having children. This discovery emphasizes the need for a holistic One Health approach that prioritizes the health and well-being of both humans and their pets. When it comes to pet owners evaluating their pets’ body condition, underestimation proved to be the predominant misperception. Addressing this issue requires comprehensive education to empower owners to recognize and comprehend their pets’ overweight status, a critical step for the overall well-being of companion animals.

## 1. Introduction

Obesity is a prevalent health concern among companion animals, particularly dogs and cats, and has been associated with a multitude of adverse health effects [1]. For instance, within European veterinary settings, overweight conditions (defined as body condition score (BCS) > 5/9 or >3/5 [2]) affect 46.6% of dogs in Denmark [3] and 45% of cats in Sweden [4]. Overweight pets are at an increased risk of developing chronic conditions such as diabetes [5], cardiovascular diseases [6], and osteoarthritis [7], and a reduced lifespan overall [8,9]. Addressing this issue requires the active involvement of pet owners, who play a crucial role in managing their pets’ body condition and weight.

One significant challenge in tackling pet obesity is the misperception of body condition by pet owners [10,11]. These misperceptions can hinder effective weight management strategies, leading to a lack of appropriate intervention and perpetuating the problem of obesity in companion animals. Few studies conducted worldwide have shed light on the global misperception of body condition by pet owners [11,12,13]. These investigations have identified some factors associated with misperception, including the animal’s body condition being either over- or underconditioned [12,13], young age [13], the living place of the owners (with people from countryside more often underestimating their dogs’ body condition score) [12], or their gender (with women better estimating their dogs’ body condition [13]). However, while these studies have contributed valuable insights into the broader problem, a specific examination of French pet owners’ misperception has not been carried out since 2006 [14,15]. This absence of recent data becomes increasingly relevant in the context of significant societal shifts, such as the rise and proliferation of the body positivity movement. This movement, championing self-acceptance despite overweight or obesity, has gained momentum through social media and may potentially limit the effectiveness of public health campaigns [16]. Conversely, the COVID-19 pandemic has led to heightened concern among Americans about overweight issues and a greater willingness to seek interventions [17]. To the authors’ knowledge, only one European study on dog owners’ perception of body condition [11] has been published since the pandemic. None have been published since then on cat owners’ body condition perceptions. This study, conducted in France, holds the potential to provide insights that could extend to other countries. 

Based on previous research, the authors hypothesize that a substantial proportion of French pet owners will demonstrate a misperception of their dogs’ and cats’ body condition, underestimating their pets’ weight status. It is expected that owners of dogs will be more likely to underestimate their dogs’ body condition than cat owners [12]. Therefore, the first aim of this study was to evaluate the owners’ perception of their pets’ body condition in France. The second aim was to compare the perception of owners when using a verbal scale versus a more comprehensive nine-point body condition score (BCS) system with accompanying figures. A third aim was to investigate the factors associated with an underestimation of the body condition. 

## 2. Materials and Methods

Pet owners were asked to voluntarily complete a questionnaire (Supplementary Files 1 and 2) during their pets’ vaccination visits at the University Veterinary Hospitals of Toulouse and Maisons-Alfort in France between 2020 and 2022. The questionnaire was designed to gather information on variables previously identified through a literature review to be associated with pet overweight. Given that overconditioning is a widely acknowledged factor that can lead to the underestimation of a pet’s body condition [12,13,18], it was imperative to recognize these aspects as potential confounding variables. The questionnaire was adapted from an online survey used in a previous study [19] and was modified for use during veterinary consultations. It was filled out before engaging in any discussions with the veterinary staff to minimize the potential for bias and influence. The study included healthy adult pets aged one year or older. Only data from the first visit of pets who presented multiple times were considered for analysis. During the consultation, the veterinarian assessed each pet’s body condition score with the nine-points scale BCS and weighed the animal [20]. All veterinarians involved in this study underwent comprehensive training and instruction to guarantee consistency in the evaluation process. They underwent a full day of training in each veterinary school consisting in scoring the same 30 dogs and 30 cats, ultimately reaching a consensus on the body condition scores.

In the survey questionnaire, owners were first asked to evaluate their pets’ body condition using the response options of “very skinny”, ”a bit skinny”, “ideal”, “a bit fat”, or “very fat” in French. This assessment will be referred to as “the verbal scale”. To align with the nine-point body condition score system, the following classifications were employed: “very skinny” and “a bit skinny” referred to a BCS < 4 for dogs and a BCS < 5 for cats, “ideal” referred to a BCS of 4–5 for dogs and a BCS of 5 for cats, “a bit fat” referred to a BCS of 6–7, and “very fat” referred to a BCS of 8–9 [2]. Based on these comparisons, the owners’ perception of their pets’ body condition was categorized into “underestimation”, “agreement”, or “overestimation” [12]. Owners were requested to assess their pets’ BCS on a scale of 1 to 9 using images from the Nestlé PURINA® Body Condition System without palpation. While this scale is typically designed for evaluating visible and palpable characteristics [20], in this instance, it was exclusively used for its visual representations [12]. This approach aimed for a swift assessment by owners, with the potential consideration of its effectiveness for application in larger studies if proven successful. This assessment, referred to as “the visual scale”, occurred after the verbal scale evaluation.

All statistical analyses were conducted using R 4.2.2. The level of agreement between verbal and visual estimations from the owners, and BCS estimations by the veterinarian were evaluated using the linear weighted Kappa (Kp) test [12]. This test assesses whether the observed agreement is higher than what would be expected by chance alone. The degree of agreement was classified as follows: very low (κ < 0.00), low (0.00 to 0.20), reasonable (0.21 to 0.40), moderate (0.41 to 0.60), high (0.61 to 0.80), and almost perfect (0.81 to 1.00) [12]. A chi-square test of proportion equality was employed to examine potential differences in perception between dog owners and cat owners [12]. For the analysis of factors associated with misperception, the number of owners who overestimated their pets’ body condition was insufficient for inclusion in the analysis, consisting of only 17 dog owners and 34 cat owners. Consequently, the study focused exclusively on examining factors associated with underestimation. For each species (dogs and cats), the associations between underestimation and explanatory variables were assessed using binary logistic regression. A stepwise backward and forward elimination process was conducted, involving the inclusion of only variables that were deemed relevant based on a literature review and had a *p*-value less than 0.25 in univariate analysis [13]. The variables considered included household size, presence of children, owner age, animal’s sex, overall activity level, access to outdoor space, presence of another dog, presence of a cat, use of a slow-feeding bowl, place of purchase of food, begging behavior, proportion of dietary energy from treats, body condition, and age for dogs and presence of children, neutering status, frequency of wet food consumption, feeding ad libitum, and body condition for cats. The resulting models were validated using the Area Under the Curve (AUC) metric [13].

## 3. Results

A total of 304 dogs and 270 cats were included in this study. A detailed description of the dogs’ and cats’ population, categorized based on the owners’ perception, can be found in Supplementary File 3.

### 3.1. Agreement between Owners and Veterinarians

Approximately 21% of dog owners and 39% of cat owners inaccurately estimated their pets’ weight in kilograms with a ±10% accuracy allowance. This included underestimation rates of 14% for dogs and 21% for cats, along with overestimation rates of 7% for dogs and 18% for cats.

When using the verbal scale, a substantial proportion of dog and cat owners disagreed with the veterinarian regarding their pets’ body condition. Among dog owners, 32.6% disagreed (27.0% underestimating and 5.6% overestimating), while among cat owners, 36.7% disagreed (24.1% underestimating and 12.6% overestimating). This percentage of disagreement increased as the excess body weight of the pets increased. Specifically, when considering pets with ideal body conditions, 12% of dog owners and 29% of cat owners disagreed with the veterinarian. For overweight, but not for obese, pets, the disagreement rate rose to 66% for dog owners and 64% for cat owners. For obese pets, all dog owners and 83% of cat owners disagreed with the veterinarian regarding their body condition (Tables S1 and S2). 

In contrast, when showing the nine-point BCS scale, 60% of dog owners and 52% of cat owners did not guess the correct BCS for their pets. However, the nine-point BCS scale enabled a greater number of owners to identify excess body weight in their pets (Tables S3 and S4). Among dog owners, 49% detected excess body weight using the BCS scale, compared to 38% using the verbal scale. Similarly, among cat owners, 75% identified excess body weight using the BCS scale, whereas only 64% did so with the verbal scale. When analyzing misperception in dogs, it was observed that all cases of misperception in underweight dogs were underestimation, while 99% of misperception in overweight dogs were underestimation. In cats, all misperceptions were overestimation in underweight cats, and 91% of misperception in overweight cats was underestimation. Comparing misperception (underestimation and overestimation) with agreement, a significant difference (*p* < 0.004) was observed between dog and cat owners, with more overestimation in cat owners and more underestimation in dog owners (Table 1). When considering only underestimation versus agreement, the significant difference disappeared (*p* > 0.05). 

With the verbal scale, the degree of concordance between owners and the veterinarian was reasonable for dogs (Kp = 0.30, 95%CI = [0.21–0.39]) and moderate for cats (Kp = 0.47, 95%CI = [0.39–0.55]). With the visual scale, the degree of concordance was moderate for both dogs (Kp = 0.43, 95%CI = [0.34–0.51]) and cats (Kp = 0.49, 95%CI = [0.42–0.56]). 

Concerning owners’ preferences for their pets’ weight, 71% and 46% of those with overweight dogs and cats (BCS 6–7), and 29% and 14% of those with obese dogs and cats (BCS 8–9), respectively, expressed a desire to maintain their pets’ current body weight. A detailed breakdown of the owners’ preferences can be found in Table S5.

### 3.2. Factors Associated with Owners’ Underestimation of Pet Body Condition

After the literature review and univariable analysis, the variables kept for multivariable analysis were number of people in the household, presence of children, owner age, animals’ sex, time of leashed activity per week, outdoor access, presence of another dog, presence of a cat, slow-feeding bowl, food store, begging behavior, percentage of energy intake by treats, body condition, and age for dogs (Table S6) and presence of children, neutering, wet food frequency, feeding ad libitum, and body condition for cats (Table S7). Factors positively associated with underestimation of body condition by dog owners were dogs’ overweight condition (OR = 81.2; 95%CI: 27.8–288; *p* < 0.001), having a cat (OR = 2.86; 95%CI: 1.14–7.60; *p* = 0.028), and having children (OR = 2.67; 95%CI: 1.04–7.15; *p* = 0.044). Age of the dog (OR = 0.85; 95%CI: 0.74–0.96; *p* = 0.010) and having another dog (OR = 0.27; 95%CI: 0.10–0.67; *p* = 0.007) were associated with a better estimation (Table 2). The dog model had a high level of accuracy with an AUC of 0.91. Factors associated with an underestimation of body condition by cat owners were cats’ overweight condition (OR = 8.56; 95%CI: 4.15–19.3; *p* < 0.001) and having children (OR = 2.55; 95%CI: 1.24–5.36; *p* = 0.011) (Table 3). The cat model had a moderate accuracy with an AUC of 0.75.

## 4. Discussion

This study found that 32.6% of dog owners and 36.7% of cat owners disagreed with the veterinarian’s assessment of their pets’ body condition, and this disagreement increases with excess body weight. The disagreement resulted in two out of seven obese dogs and one out of seven obese cats, where the owners were content in maintaining the current weight of their pet. This included one dog owner perceiving their dog as “ideal” and another dog owner, along with a cat owner, who considered their pet to be “a bit fat”. Only 79% of dog owners and 61% of cat owners were able to tell their pets’ weight in kilograms (±10%). The degree of concordance for body condition between owners and veterinarian was reasonable (Kp = 0.30) for dogs and moderate (Kp = 0.47) for cats. The implementation of the photographs from the nine-point BCS scale did not lead to a notable improvement in the degree of concordance between owners and veterinarians. However, with this scale, a higher proportion of owners with overweight pets described their animals as being overweight. Among dog and cat owners, factors associated with the underestimation of body condition were the pets’ overweight status and having children. For dogs, underestimation was also associated with owning a cat, and an agreement was associated with the increasing age of the dog and owning another dog.

In a previous British study, 69% of dog owners were able to provide a description of their dogs’ weight in either kilograms or pounds [18]. However, the level of precision in their estimates was not specified. This finding falls between the results of the current study, with 55% of dog owners achieving a precision level of ±5% and 79% achieving a precision level of ±10%. The estimation accuracy of cat owners was relatively lower, even when considering the broader precision range of ±10%: only 61% of cat owners accurately estimated their cat’s weight within this range. 

Beyond simply knowing the weight of their pets, it is crucial for owners to be able to interpret it accurately. Unfortunately, when it comes to assessing body condition, there is a substantial level of misperception among pet owners, which aligns with the findings from previous studies showing low Kp [13,14,15]. Although the percentage of owners with dogs in an ideal body condition in agreement with the veterinarian’s assessment was similar to a previous study (here 88% vs. 80%), there is a lower agreement among owners of overweight dogs (here, 31% vs. 53%) [18]. 

Interestingly, when considering cats, owners of overweight cats exhibited higher agreement levels compared to dog owners, with 55% agreement observed. This outcome aligns with the findings from the sole prior study that directly compared the opinions from dog owners to those of cat owners [12]. The hypothesis proposed in the aforementioned study suggested that the disparity might be due to dog owners who engage in physical activities with their pets possibly perceiving exercise as a contributing factor and, consequently, underestimating their animals’ weight. Another possible explanation could be that excess body weight is more noticeable in smaller pets like cats from a human perspective, which makes it easier for owners to accurately assess their cats’ weight. In fact, it has been suggested that the larger body size of male cats might present challenges for owners when evaluating their body condition [21].

Consistent with previous studies, it has been observed that pet owners tend to normalize their pets’ body condition, leading to an underestimation in the case of overweight animals and an overestimation for underweight ones [13]. The misperception surrounding obesity has been shaped by societal factors, including a shift in generational norms regarding human body weight. What was once considered “overweight” now tends to be viewed as “about right” [22]. This shift is further amplified by the influence of social media platforms [16], and a similar trend may be occurring in the context of pets, perpetuating the belief that overweight animals are the norm in today’s society. However, it is crucial to recognize the potential of these communication channels to be harnessed for a positive purpose. Rather than perpetuating the misperception, social media platforms could serve as effective tools to fight pet obesity by disseminating advice and raising awareness about the associated risks, similar to the efforts mentioned regarding pediatric obesity [22]. When communicating about pet obesity, it is essential to be mindful of the potential for unintended consequences. Framing the issue in a way that suggests blame on pet owners may inadvertently reinforce the notion that excess weight in pets reflects low moral worth [23,24], leading owners to reject the information altogether and hindering their ability to address the problem effectively. Another explanation for this normalization is that owners, interacting with their animals every day, might not notice gradual weight gain. Weighing the pet regularly could help counteract this, as is the case in humans [25]. However, this study reveals that not all owners are necessarily aware of their pets’ weight. This may explain a lack of monitoring and a regular weight gain that goes unnoticed. It could be beneficial to encourage owners to track their pets’ weight trajectory throughout its life, such as parents for children [26].

The misperception surrounding pet body condition is a significant problem with far-reaching implications. Firstly, it contributes to the increasing prevalence of overweight animals, as an underestimation of pets’ body conditions has been associated with pets being overweight [15]. Additionally, this misperception poses a challenge when veterinarians attempt to implement weight loss plans. Owners, who play a crucial role in the process, may lack motivation [10] or may fail to notice their pets’ weight loss progress, especially if they do not regularly weigh their pets. 

This study sheds light on the complexity of weight loss plans by exploring the intentions of owners regarding their animals’ weight. Interestingly, 68% of dog owners and 44% of cat owners with overweight pets expressed a desire for their animals to maintain the same weight. It is worth noting that this question was posed prior to the consultation, and it would have been valuable to assess any change in owners’ perspectives following the discussion with the veterinarian. Disagreements between the perspectives of veterinarians and clients have been observed in previous studies [27], and research has demonstrated that many owners remain in a pre-contemplative state, even when aware of their animals’ overweight status [23]. One of the primary concerns in Western societies is the humanization of pets [28]. The act of providing food has become a means for owners to express their affection, and they often associate feeding with pleasure [10,29]. This humanization process can lead to resistance from owners regarding weight loss plans and may hinder effective communication with veterinarians [18].

Apart from excess body weight, one intriguing finding was that having children was the sole factor associated with an underestimation in both dog and cat owners, with approximately 2.5 times higher odds of underestimating their pets’ body condition. Interestingly, studies conducted in human populations have discovered a similar trend among parents in underestimating their overweight/obese child’s body condition [30,31,32]. As proposed in the context of pets, the normalization of body condition may result from unconscious habituation, given the routine exposure to their child on a daily basis, coupled with a self-preservation instinct aimed at avoiding feelings of guilt for their children’s overweight [33,34]. Several other hypotheses have been proposed to explain this underestimation phenomenon. One possibility is that parents may resist labeling or stigmatizing their children and, consequently, fail to acknowledge that their child is overweight [35,36,37]. Moreover, parents may be reluctant to recognize their child’s weight issue because addressing it would require implementing healthy lifestyle changes for themselves as well [30]. This same tendency may extend to how parents perceive their pets’ body condition. To gain deeper insights into these hypotheses, an intriguing approach would be to conduct a study where parents are asked to rate the body condition of both pets and children who are not their own. This method draws inspiration from a previous experiment in which adults were asked to assess images of men, revealing a tendency to underestimate the status of overweight and obese individuals [38]. While employing children in such a study necessitates stringent ethical approval, this approach would provide a deeper understanding of whether parental bias extends beyond their immediate family. Another explanation could be that parents are preoccupied with their children’s needs and may not prioritize monitoring their pets’ body condition as closely. Moreover, parents may unconsciously use their children’s body condition as a reference point for evaluating their pets, even though there are notable differences. Human babies, in particular, have a higher proportion of body fat compared to many other species, rendering them an unsuitable benchmark [39]. As a result, humans may perceive the plumper appearance of pets as “cute”, much like how they find obese humans endearing because curvilines can make them resemble babies [39].

In any case, the observation that parents fail to perceive their children and pets as overweight provides compelling evidence for the importance of adopting a “One Health” approach [40]. Considering this shared concern, collaboration between veterinarians and pediatricians holds immense potential for developing joint awareness campaigns aimed at addressing overweight issues in children and pets alike. This collaboration can lead to impactful interventions that promote healthy habits, raise awareness, and ultimately contribute to improving the well-being of both children and pets.

Findings of this study suggest that dog owners who also have cats may have a higher likelihood of underestimating their dogs’ body condition compared to dog owners without cats. Further research and investigation would be needed to understand the underlying reasons for this association. Factors such as caregiving patterns, or interactions between the two species could potentially contribute to this phenomenon. 

Age (in years) was identified as a factor associated with a better estimation of body condition by dog owners. This finding is consistent with a previous study which reported a 24% reduced risk of underestimation in senior dogs aged 9–18 years [13]. The hypothesis previously mentioned is that owner attitudes towards acceptable body shape may evolve over a dog’s lifespan. Another plausible explanation is that owners tend to be more attentive and to provide greater care for their older pets, knowing that these animals are susceptible to illness and potential weight loss. This result could also highlight that older dogs may be more likely to have owners with a better understanding of dogs in general and ideal body condition. Furthermore, owning another dog was also found to be associated with an agreement in our study. This finding reinforces the “knowledge hypothesis” as owners of multiple dogs may possess a broader awareness of canine body condition, potentially contributing to more accurate assessments. In a prior study, the previous ownership of dogs in individuals’ lives was linked to a disagreement about their actual dogs’ body condition [11]. However, in this current study, participants were not queried about their historical dog ownership; instead, they were asked to provide the current number of dogs in their care. It may be that possessing multiple dogs may potentially improve owners’ perception by enabling comparisons among them. Indeed, research indicates that the perception of human body condition often involves a comparative analysis with others [41]. These results underscore the possibility of owners’ perception being transformed and enhanced through education over time. 

Although previous studies did not demonstrate a superior agreement coefficient for visual scales compared to verbal scales [42], it remains a valuable tool in raising awareness among owners of overweight pets about their pets’ weight issues, as observed in both the current study and previous studies conducted in France [14,15]. In this study, the scale was used solely for pictures and descriptions, with owners not encouraged to perform palpation. The aim was to be able to implement this quick assessment in larger studies. In a recent study in Sweden, instructing owners in the proper utilization of the nine-point BCS scale, including palpation, was significantly associated with an improvement in their assessment [11]. This led to no difference in the mean estimated BCS between owners and veterinary staff, demonstrating a more substantial improvement compared to studies, such as this one, using only pictures [43]. Taken altogether, these results affirm the necessity of teaching owners palpation. Veterinarians should provide owners the opportunity to train with tactile models simulating various BCS. This hands-on approach could help owners identify different BCS levels by feeling the models, offering a more effective alternative to waiting room posters. Future studies should concentrate on validating the use of online video tutorials to teach BCS estimation with pictures and palpation to owners. This approach could be quicker than direct teaching by veterinary staff and more beneficial than relying solely on pictures.

This study has several limitations that should be considered. Firstly, the assessment of BCS was conducted by six veterinarians, which could introduce some variability in the measurements. However, efforts were made to ensure consistency in scoring by providing a full day of training to vet staff in each school and adhering to the standardized guidelines of the validated nine-point scale BCS method, known for its good reproducibility [20]. Secondly, it is important to acknowledge that the study was conducted in two specific veterinary schools, namely Alfort and Toulouse. Consequently, the sample of participants may not be fully representative of the entire population of dog and cat owners in France. However, the study offers valuable insights into the perception of body condition among owners of healthy pets and can be compared to other studies also made in veterinary clinics. In contrast to a preceding study conducted in a Brazilian veterinary clinic from 2013 to 2018, this study demonstrates higher levels of agreement for owners of both dogs (67.4% vs. 38.8%) and cats (63.3% vs. 40.9%) [12]. The level of agreement may potentially be higher in European countries compared to Brazil, as indicated by another European study conducted in Glasgow, which reported a 55.9% agreement rate for dog owners [13]. Finally, owners were not taught the palpation evaluation of the pets’ BCS, hindering the ability to assess potential improvements in their estimations

Acknowledging its limitations, this study contributes valuable insights into owners’ perception of pet body condition and highlights areas for further research. For instance, future studies should consider evaluating the attachment between owners and their pets, possibly using tools like the Lexington Attachment to Pet Scale [44], even though its validation in the French language is lacking. This assessment could shed light on the role of the animal within the family unit. Furthermore, incorporating specific questions related to whether owners view their pets as children could provide valuable insights into the connection between having children and underestimating the pets’ weight. Additionally, future studies should encompass inquiries about prior veterinary consultations, including whether the veterinarian discussed the pet’s weight, and introduce a post-consultation questionnaire for a comprehensive understanding of the owner–veterinarian interaction in weight management.

## 5. Conclusion

Misperception of pets’ body condition by owners was estimated to be about 33% for dogs and 37% for cats in France between 2020 and 2022. This study unveiled a significant association between having children and the tendency to underestimate their pets’ body condition. This noteworthy finding underscores the importance of future research to delve deeper into this connection, further contributing to a One Health approach that considers the health and well-being of both humans and their animal companions. The most common form of misperception was underestimation, leading to owners being satisfied with the weight of their overweight pets. To address this issue, owners can be educated to recognize and understand the overweight status of their pets. Since using pictures of different BCS levels has not demonstrated significantly greater effectiveness, this education should emphasize the technique of palpation for accurate BCS estimation.

## Figures and Tables

**Table 1:** Differences between cat and dog owners’ perception of their pets’ body condition.

Characteristic	Underestimation	Agreement	Overestimation	*p*-Value ^1^
Species				**0.004**
cat	65 (43%)	171 (46%)	34 (69%)	
dog	85 (57%)	204 (54%)	15 (31%)	

^1^ Chi-square test. Statistically significant *p*-values (*p* < 0.05) are shown in bold.

**Table 2:** Results of the multivariable analysis for owners’ underestimation of dog body condition.

Variable	N	Odds Ratios	CI ^1^	*p*-Value
Dog’s status (Overweight)	82	81.2	27.8–288	**<0.001**
Dog’s age (Years)	226	0.85	0.74–0.96	**0.010**
Dog’s sex (Male)	111	1.94	0.82–4.89	0.14
Another dog (Yes)	76	0.27	0.10–0.67	**0.007**
Cat (Yes)	68	2.86	1.14–7.6	**0.028**
Children (Yes)	58	2.67	1.04–7.15	**0.044**

^1^ Confidence Interval. Statistically significant *p*-values (*p* < 0.05) are shown in bold.

**Table 3:** Results of the multivariable analysis for owners’ underestimation of cat body condition.

Variable	N	Odds Ratios	CI ^1^	*p*-Value
Status (Overweight)	123	8.56	4.15–19.3	**<0.001**
Children (Yes)	65	2.55	1.24–5.36	**0.011**

^1^ Confidence Interval. Statistically significant *p*-values (*p* < 0.05) are shown in bold.

## Data Availability

All the datasets analyzed throughout the present study are available from the corresponding author on reasonable request.

## Supplementary Materials

The following supporting information can be downloaded at https://www.mdpi.com/article/10.3390/ani13233646/s1, Supplementary File 1: Questionnaire for dog owners; Supplementary File 2: Questionnaire for cat owners; Supplementary File 3: Description of the population; Table S1: Dog owners’ opinion according to BCS rated by a veterinarian; Table S2: Cat owners’ opinion according to BCS rated by a veterinarian; Table S3: Dog owners’ BCS with the visual scale according to BCS rated by a veterinarian; Table S4: Cat owners’ BCS with the visual scale according to BCS rated by a veterinarian; Table S5: Owners’ will about their pets’ weight; Table S6: Results of the univariate analysis for dog owners’ underestimation; Table S7: Results of the univariate analysis for cat owners’ underestimation.
