# Supplementary material for: The Perception of the Body Condition of Cats and Dogs by French Pet Owners and the Factors Influencing Underestimation
Source: Animals (Basel). 2023 Nov 25;13(23):3646. doi: 10.3390/ani13233646 (PMC10705725; doi:10.3390/ani13233646)
Supplement: Supplementary file 1 [file animals-13-03646-s001.zip › Supplementary File 1.pdf]

1- Does this dog living with you ?
   a. Yes
   b. No
2- Did you already filled this questionnaire since january 2020 ?
   a. No
   b. Yes for this dog
   c. Yes for another dog
3- If yes for another dog : Name of the dog : ______________________
4- Postal code : ________________________
5- Number of person in the household (you included) : __________________
6- Number of children in the household : ______________________
7- Your age :
   a. 18-25 years old
   b. 26-40 years old
   c. 41-60 years old
   d. > 60 years old
8- If appropriate, age of your compagnon :
   a. 18-25 years old
   b. 26-40 years old
   c. 41-60 years old
   d. > 60 years old
9- Your profession : ____
10- If appropriate, the profession of your companion : ____________________________________
11- Birth's date of your dog : __________________________
12- Your dog is :
   a. A neutered male
   b. A entire male
   c. A neutered female
   d. A entire female
13- If applicable, is your female dog in gestation ?
   a. Yes
   b. Non
14- If yes, since how many days ?
15- Age of the neutering
   a. Before 8 months
   b. Between 8 and 12 months
   c. Between 1 and 2 years
   d. Between 3 and 7 years
   e. After 7 years
   f. I don't know
16- Is your dog a pure breed ?
   a. Yes
   b. No
17- Breed of the dog : ___________________________
18- Inscription on the LOF (Book of French origins)
19- Type of hair
   a. Naked

b. Short
c. Mi-long
d. Long

20- Color the the hair :____________________

21- Muzzle :
a. Mashed
b. Normal
c. Prounouced stop
d. Non-prounouced stop

22- Chronical disease ?
a. Yes
b. Non

23- If yes, which one : _________________________

24- Medication ?
a. Yes
b. Non

25- If yes, which ones : ________________________

26- Your dog is
a. Very skinny
b. Skinny
c. Normal
d. A bit fat
e. Very fat

27- With the help of the chart at the end of this document, which number corresponds to your dog? _____________

28- You want your dog
a. Keep this weight
b. Lose weight
c. Gain weight

29- In the 12 months, how many time do you have weighted your dog?
a. None
b. Once
c. More than once

30- Place to weigh the dog
a. I do not weight my dog
b. At home
c. At the veterinarian clinic
d. Other

31- Weight change ?
a. Non
b. Yes, he has gained weight
c. Yes, he has lost weight

32- Deworming ?
a. Yes, each 2 months and more
b. Yes, each 3 months
c. Yes, each 6 months
d. Yes, each years
e. Sometimes

f. Never

33- Weight of the dog : ____

34- On a scale of 1 to 10, how active would your dog be? Take into account the game, alone or with you, and its behavior outside if necessary.

35- Do you find your dog to be well muscled?
  a. Yes
  b. No

36- What is the off-leash walking time per week? (outside the confinement period)
  a. Less than 1 hour
  b. 2 to 4h
  c. 4h to 6h
  d. More than 6h

37- What is the leash walking time per week? (outside the confinement period)
  a. Less than 1 hour
  b. 2 to 4h
  c. 4h to 6h
  d. More than 6h

38- Does your dog exercise for sports? If yes which ?
  a. None
  b. Agility
  c. Sled/race
  d. Guide dog
  e. Guard
  f. Hunting
  g. Other

39- If your dog is doing an activity, how many days per week does it represent? : _________________________________

40- On a scale of 1 to 10, how active would you say your dog was ONE YEAR AGO?

41- Type of habitat
  a. Appartement
  b. House
  c. Other

42- What is the interior surface in m² accessible to the dog? ___________________

43- Does the dog have to go up and down stairs?
  a. Yes
  b. No

44- Does your dog have free access to:
  a. Terrace
  b. Kennel
  c. Garden
  d. No access to outside

45- How often do you take your dog out (outside the garden and home)?
  a. Every day, once
  b. Every day, several times a day
  c. Sometime (i.e, the week-end)
  d. Rarely (i.e, during holiday)

46- If your dog lives part of the year in the city and part in the countryside, what time does he spend, in %, in the countryside? (If he only lives in town, put: 0) :________________

47- How long does the dog go out per day? (outside the confinement period)
   a. Less than 30 min
   b. Between 30 and 60 min
   c. Between 1h and 2 h
   d. More than 2h
48- Do you or a member of your family play with the dog?
   a. Yes, each day, once
   b. Yes, each day, several time a day
   c. Yes, some time
   d. Yes, rarely
   e. Non
49- If you play with your dog, which type of play ? : ___________________________________
50- Do you have other animals in contact with your dog?
   a. Yes
   b. No
51- Number of dogs (including the present dog) :_________________
52- Number of cats :___________________________
53- Number of rabbits :____________________
54- Nimber of others animals (birds, snake, ferrets, ….) :__________________
55- Interaction of your dogs with your others animals

	Each day	Often	Rarely	Sometime	Never
He plays with it and the game session is accepted by both					
He plays with it but the dog is forced					
He plays with it but the other animal doesn't want					
He sleeps with					

56- Does your dog share spaces or objects with other pets in the house?
   a. Yes, his water bowls
   b. Yes, his food bowls
   c. Yes, his toys
   d. Yes, his sleeping
   e. Nothing of the sort
57- Is this sharing going well? (Choose the option that corresponds to the most common situation.)
   a. No, my dog refuses the interaction
   b. No, the other animal refuses the interaction
   c. Yes
   d. My dog and my other animals never cross paths

58- Does your dog have toys?
   a. Yes, but he doesn't use it
   b. Yes, and he uses it
   c. No
59- Where does your dog primarily sleep?
   a. Outside in a doghouse or kennel
   b. Inside where he wants
   c. In the garage
   d. Inside in a reserved place (under stairs, ...)
   e. Inside, in the living room
   f. Inside, in the kitchen
   g. Inside, in a room
   h. Other
60- How is his sleep area ?
   a. In height
   b. A carpet
   c. A basket
   d. A sofa
   e. A reserved bed
   f. In your bed
   g. In your child's bed
   h. Other
61- What type of bowl does he have?
   a. A classic bowl
   b. An "anti-glutton" bowl
   c. A distributor
   d. An electronic / automatic bowl
   e. Other
62- Does your dog have toys to split or slow down food intake?
   a. Yes but he does not use it
   b. Yes and he uses it
   c. No
63- What type of food do you primarily feed your dog?
   a. Industrial food (croquettes, boxes or bags)
   b. Home-made ration (BARF, classic home made ration, industrial BARF, Whole prey, ...)
   c. A mixture of both (for example, 50% kibble and 50% homemade ration)

IN CASE OF INDUSTRIAL FOOD :

64- What is your dog's main type of diet (everyday) ?
   a. Complete dry food (kibbles)
   b. Complete food in box or sachet
65- How much do you distribute each day?
   a. An amount prescribed by the veterinarian
   b. A quantity indicated by the food manufacturer (on the back of the bag for example)
   c. A quantity indicated by someone other than the manufacturer or the veterinary team
   d. My dog has unlimited food and eats according to his appetite

66- For kibbles, this quantity is distributed in how many meals?
   a. Self-service (as soon as there is no more food, the dog is served again)
   b. Once a day
   c. Twice a day
   d. More than twice a day
   e. I never give dry food

67- For food in cans or sachets, this quantity is distributed in how many meals?
   a. Self-service (as soon as there is no more food, the dog is served again)
   b. Once a day
   c. Two to four times a day
   d. More than four times a day
   e. I never give wet food

68- Where do you usually buy the main food? (outside the confinement period)
   a. In a garden center or specialized store
   b. In a veterinarian clinic
   c. On the Internet
   d. It depends (on promotions, ...)
   e. Other

69- To which category (ies) does the main food belong? (many possible responses)
   a. Generic food (food for all types of dogs)
   b. Growth or pediatric
   c. Adult
   d. Senior
   e. For neutered dog
   f. Light
   g. Dietetic
   h. No grain/no gluten
   i. Vegetarian diet
   j. Primitive
   k. Other

70- In the case of a dietetic food, for which condition is it?
   a. Urinary/renal
   b. Hepatic/pantreatic
   c. Diebetic
   d. Skin or ostheoatrhitis
   e. Other

IN CASE OF HOMEMADE RATION

71- For this ration, how much do you distribute each day?
   a. A prescribed quantity
   b. A free amount according to my dog's appetite
   c. Other

72- How do you split the distribution of this ration?
   a. Once
   b. Two to four times a day
   c. More than four times a day

73- Does the homemade ration belong to one of these categories?
   a. BARF

b. Whole prey/prey
c. Vegetarian
d. No grain (except BARF)
e. No

74- How do you make the recipe?
   a. Prescribed by a veterinarian in consultation
   b. Prescribed by a veterinarian, online
   c. Personal recipe
   d. Recipe found in a book written by a veterinarian
   e. Recipe found on the internet (facebook group) or in a book but not developed by a veterinarian

75- Does your recipe include food supplements with minerals and vitamins?
   a. Yes
   b. No

76- If yes, which one ? _______________________________

IN CASE OF MIX INDUSTRIAL+HOMEMADE RATION

77- Please give details of your animal's daily ration (amount of dry food, amount of wet food, amount of table scraps and household food):

	Industrial dry food	Industrial wet food	Home made ration	leftovers
Quantity (g or %)				

IN ALL CASES

78- Distribute in addition:
   a. Table scraps, at the table
   b. Table scraps, in the bowl
   c. Nothing of the sort

79- Do you distribute rewards?
   a. Yes
   b. No

80- If yes, when ?
   a. Education
   b. Work
   c. Play
   d. When you want

81- If yes, which one and which quantity per day ?

82- Where does the meal take place?
   a. In the kitchen
   b. When the family eat
   c. Alone
   d. On his sleeping place
   e. In his kennel
   f. Outside
   g. Other

83- How many people are likely to feed the dog (usually)?______________________________

84- Are the quantities measured systematically?

  a. Systematic weighing
  b. Rigorous use of a measuring cup (line with the marker for example)
  c. Approximate use of a measuring cup
  d. Estimate according to the duration of the package
  e. No

85- Does your dog eat better in the presence of another dog?

  a. Yes
  b. No
  c. I don’t know

86- If yes, If so, when he is in a group compared to when he is alone does he finish his bowl ...

  a. Faster
  b. Slower
  c. Always the last one

87- Ingestion speed

  a. Normal
  b. Slow
  c. Fast or very fast

88- By submitting this form, I agree that the information entered will be used as part of the study carried out by the National Veterinary Schools of Alfort and Toulouse

  a. Yes
  b. No
