# Supplementary material for: The Perception of the Body Condition of Cats and Dogs by French Pet Owners and the Factors Influencing Underestimation
Source: Animals (Basel). 2023 Nov 25;13(23):3646. doi: 10.3390/ani13233646 (PMC10705725; doi:10.3390/ani13233646)
Supplement: Supplementary file 1 [file animals-13-03646-s001.zip › Supplementary File 2.pdf]

1- Does this cat living with you ?
   a. Yes
   b. No
2- Did you already filled this questionnaire since january 2020 ?
   a. No
   b. Yes for this cat
   c. Yes for another cat
3- If yes for another cat : Name of the cat : ______________________
4- Postal code : ________________________
5- Number of person in the household (you included) : __________________
6- Number of children in the household : ______________________
7- Your age :
   a. 18-25 years old
   b. 26-40 years old
   c. 41-60 years old
   d. > 60 years old
8- If appropriate, age of your compagnon :
   a. 18-25 years old
   b. 26-40 years old
   c. 41-60 years old
   d. > 60 years old
9- Your profession : ____
10- If appropriate, the profession of your companion : ____________________________________
11- Birth's date of your cat : __________________________
12- Your cat is :
   a. A neutered male
   b. An entire male
   c. A neutered female
   d. An entire female
13- If applicable, is your female cat in gestation ?
   a. Yes
   b. Non
14- If yes, since how many days ?
15- Age of the neutering
   a. Before 8 months
   b. Between 8 and 12 months
   c. Between 1 and 2 years
   d. Between 3 and 7 years
   e. After 7 years
   f. I don't know
16- Is your cat a pure breed ?
   a. Yes
   b. No
17- Breed of the cat : ___________________________
18- Type of hair
   a. Naked
   b. Short

   c. Mi-long
   d. Long
19- Color the the hair :____________________
20- Muzzle :
   a. Mashed
   b. Normal
   c. Pointed
21- Chronical disease ?
   a. Yes
   b. Non
22- If yes, which one : _________________________
23- Medication ?
   a. Yes
   b. Non
24- If yes, which ones : ________________________
25- Your cat is
   a. Very skinny
   b. Skinny
   c. Normal
   d. A bit fat
   e. Very fat
26- In your opinion, what's the weight (in kg) of your cat ? _________
27- With the help of the chart at the end of this document, which number corresponds to your cat? _____________
28- You want your cat
   a. Keep this weight
   b. Lose weight
   c. Gain weight
29- In the 12 months, how many time do you have weighted your cat?
   a. None
   b. Once
   c. More than once
30- Place to weigh the cat
   a. I do not weight my cat
   b. At home
   c. At the veterinarian clinic
   d. Other
31- Weight change ?
   a. No
   b. Yes, he has gained weight
   c. Yes, he has lost weight
32- Deworming ?
   a. Yes, each 2 months and more
   b. Yes, each 3 months
   c. Yes, each 6 months
   d. Yes, each years
   e. Sometimes
   f. Never

33- On a scale of 1 to 10, how active would your cat be? Take into account the game, alone or with you, and its behavior outside if necessary.
34- Do you find your cat to be well muscled?
   a. Yes
   b. No
35- On a scale of 1 to 10, how active would you say your cat was ONE YEAR AGO?
36- Type of habitat
   a. Appartement
   b. House
   c. Other
37- What is the interior surface in m² accessible to the cat? ____________________
38- Does your cat have free access to:
   a. Terrace
   b. Kennel
   c. Garden
   d. No access to outside
39- How often do you take your cat go out (outside terrace or balcony)?
   a. Every day, once
   b. Every day, several times a day
   c. Sometime (i.e, the week-end)
   d. Rarely (i.e, during holiday)
40- Do you have other animals in contact with your cat?
   a. Yes
   b. No
41- Number of cats (including the present cat) :_________________
42- Number of cats :___________________________
43- Number of rabbits :____________________
44- Number of others animals (birds, snake, ferrets, ….) :__________________
45- Interaction of your cats with your others animals

	Each day	Often	Rarely	Sometime	Never
He plays with it and the game session is accepted by both					
He plays with it but the cat is forced					
He plays with it but the other animal doesn't want					
He sleeps with					

46- Does your cat share spaces or objects with other pets in the house?
   a. Yes, his water bowls
   b. Yes, his food bowls

  c. Yes, his toys
  d. Yes, his sleeping
  e. Yes, his cat tree
  f. Nothing of the sort

47- Is this sharing going well? (Choose the option that corresponds to the most common situation.)
  a. No, my cat refuses the interaction
  b. No, the other animal refuses the interaction
  c. Yes

48- Does your cat spend a lot of time up high?
  a. Yes
  b. No, he doesn't like
  c. No, he's not allowed

49- Does your cat have one or more cat trees, shelves or other perching systems?
  a. Yes, only one
  b. Yes, several
  c. No, or he doesn't use them

50- Where is located his food bowl?
  a. High
  b. On the ground
  c. Next to his litterbox
  d. Far from his litterbox

51- What kind of feed bowl does he have?
  a. Classic
  b. Anti-glutton
  c. Dispenser
  d. A connected bowl
  e. Other: ________

52- Does your cat have toys which dispense food?
  a. Yes, and he uses them
  b. Yes, but he doesn't use them
  c. No

53- Does your cat have toys?
  a. Yes, but he doesn't use it
  b. Yes, and he uses it
  c. No

54- Where does your cat primarily sleep?
  a. Outside
  b. Inside in the home
  c. Inside but in a garage or barn
  d. Other

55- What type of food do you primarily feed your cat?
  a. Industrial food (croquettes, boxes or bags)
  b. Home-made ration (BARF, classic home made ration, industrial BARF, Whole prey, ...)
  c. A mixture of both (for example, 50% kibble and 50% homemade ration)

IN CASE OF INDUSTRIAL FOOD :

56- What is your cat's main type of diet (everyday) ?
  a. Complete dry food (kibbles)
  b. Complete food in box or sachet

57- How much do you distribute each day?
  a. An amount prescribed by the veterinarian
  b. A quantity indicated by the food manufacturer (on the back of the bag for example)
  c. My cat has unlimited food and eats according to his appetite

58- For kibbles, this quantity is distributed in how many meals?
  a. Self-service (as soon as there is no more food, the cat is served again)
  b. Once a day
  c. Twice a day
  d. More than twice a day
  e. I never give dry food

59- For food in cans or sachets, this quantity is distributed in how many meals?
  a. Self-service (as soon as there is no more food, the cat is served again)
  b. Once a day
  c. Two to four times a day
  d. More than four times a day
  e. I never give wet food

60- Where do you usually buy the main food?
  a. In a garden center or specialized store
  b. In a veterinarian clinic
  c. On the Internet
  d. It depends (on promotions, ...)
  e. Other

61- To which category (ies) does the main food belong? (many possible responses)
  a. Generic food (food for all types of cats)
  b. Growth or pediatric
  c. Adult
  d. Senior
  e. For neutered cat
  f. Light
  g. Dietetic
  h. No grain/no gluten
  i. Vegetarian diet
  j. Primitive
  k. Other

62- In the case of a dietetic food, for which condition is it?
  a. Urinary/renal
  b. Hepatic/pantreatic
  c. Diebetic
  d. Skin or ostheoatrhitis
  e. Other : ______________________

IN CASE OF HOMEMADE RATION

63- For this ration, how much do you distribute each day?
  a. A prescribed quantity
  b. A free amount according to my cat's appetite

c. Other

64- How do you split the distribution of this ration?
   a. Once
   b. Two to four times a day
   c. More than four times a day
65- Does the homemade ration belong to one of these categories?
   a. BARF
   b. Whole prey/prey
   c. Vegetarian
   d. No grain (except BARF)
   e. No
66- How do you make the recipe?
   a. Prescribed by a veterinarian in consultation
   b. Prescribed by a veterinarian, online
   c. Personal recipe
   d. Recipe found in a book written by a veterinarian
   e. Recipe found on the internet (facebook group) or in a book but not developed by a veterinarian
67- Does your recipe include food supplements with minerals and vitamins?
   a. Yes
   b. No
68- If yes, which one ? ________________________________

IN CASE OF MIX INDUSTRIAL+HOMEMADE RATION

69- Please give details of your animal's daily ration (amount of dry food, amount of wet food, amount of table scraps and household food):

	Industrial dry food	Industrial wet food	Home made ration	leftovers
Quantity (g or %)				

IN ALL CASES

70- Distribute in addition:
   a. Table scraps, at the table
   b. Table scraps, in the bowl
   c. Nothing of the sort
71- Do you distribute rewards?
   a. Yes
   b. No
72- Ingestion speed
   a. Normal
   b. Slow
   c. Fast or very fast
73- By submitting this form, I agree that the information entered will be used as part of the study carried out by the National Veterinary Schools of Alfort and Toulouse
   a. Yes
   b. No
