# Supplementary material for: The Perception of the Body Condition of Cats and Dogs by French Pet Owners and the Factors Influencing Underestimation
Source: Animals (Basel). 2023 Nov 25;13(23):3646. doi: 10.3390/ani13233646 (PMC10705725; doi:10.3390/ani13233646)
Supplement: Supplementary file 1 [file animals-13-03646-s001.zip › Supplementary File 3.pdf]

DOGS

**Characteristic**	**Underestimation**, N = 85	**Agreement**, N = 204	**Overestimation**, N = 15
Sex			
Female	49 (58%)	93 (46%)	6 (40%)
Male	36 (42%)	111 (54%)	9 (60%)
Neutered [Yes]	47 (55%)	110 (54%)	7 (47%)
Sex and neutering			
Female	18 (21%)	33 (16%)	2 (13%)
Male	20 (24%)	61 (30%)	6 (40%)
Neutered female	31 (36%)	60 (29%)	4 (27%)
Neutered male	16 (19%)	50 (25%)	3 (20%)
Purebreed [Yes]	66 (78%)	148 (74%)	12 (80%)
Unknown	0	5	0
Age category			
Adult	49 (58%)	132 (65%)	9 (60%)
Senior	24 (28%)	47 (23%)	5 (33%)
Geriatric	12 (14%)	25 (12%)	1 (6.7%)
Hairs			
Short	37 (44%)	92 (46%)	6 (40%)
Half_long	34 (40%)	78 (39%)	6 (40%)
Long	14 (16%)	30 (15%)	3 (20%)
Unknown	0	4	0
Status			
Underweight	3 (3.5%)	2 (1.0%)	0 (0%)
Ideal	9 (11%)	168 (82%)	14 (93%)
Overweight	66 (78%)	34 (17%)	1 (6.7%)
Obese	7 (8.2%)	0 (0%)	0 (0%)
9-points scale BCS by veterinarian			
3	3 (3.5%)	2 (1.0%)	0 (0%)
4	5 (5.9%)	17 (8.3%)	0 (0%)
5	4 (4.7%)	151 (74%)	14 (93%)
6	56 (66%)	19 (9.3%)	0 (0%)
7	10 (12%)	15 (7.4%)	1 (6.7%)
8	5 (5.9%)	0 (0%)	0 (0%)
9	2 (2.4%)	0 (0%)	0 (0%)

Dogs’ characteristics

**Characteristic**	**Underestimation**, N = 85	**Agreement**, N = 204	**Overestimation**, N = 15
Owner age			
< 40	54 (64%)	144 (71%)	9 (60%)
≥ 40	30 (36%)	58 (29%)	6 (40%)
Unknown	1	2	0
Retired [Yes]	15 (19%)	27 (14%)	3 (20%)
Unknown	4	11	0
Owner perception			
Skinny	12 (14%)	0 (0%)	0 (0%)
Optimal	67 (79%)	170 (83%)	0 (0%)
A bit fat	6 (7.1%)	34 (17%)	14 (93%)
Very fat	0 (0%)	0 (0%)	1 (6.7%)
BCS by owner			
1	1 (1.5%)	1 (0.7%)	0 (0%)
2	1 (1.5%)	0 (0%)	0 (0%)
3	7 (11%)	9 (6.0%)	0 (0%)
4	2 (3.1%)	12 (8.1%)	1 (6.7%)
5	37 (57%)	100 (67%)	6 (40%)
6	4 (6.2%)	7 (4.7%)	1 (6.7%)
7	11 (17%)	18 (12%)	7 (47%)
8	1 (1.5%)	1 (0.7%)	0 (0%)
9	1 (1.5%)	1 (0.7%)	0 (0%)
Unknown	20	55	0
Owner’s preference			
Make the dog gain weight	10 (12%)	6 (3.0%)	0 (0%)
Make the dog maintain that weight	64 (77%)	166 (84%)	7 (47%)
Make the dog lose weight	9 (11%)	25 (13%)	8 (53%)
Unknown	2	7	0
Number of weighing since last year			
None	8 (9.5%)	19 (9.5%)	0 (0%)
One	39 (46%)	82 (41%)	5 (33%)
Several	37 (44%)	99 (50%)	10 (67%)
Unknown	1	4	0
Weighing location			
Never weighed	3 (3.7%)	9 (4.5%)	0 (0%)
Home	16 (20%)	52 (26%)	6 (40%)
Vet	63 (77%)	139 (70%)	9 (60%)
Unknown	3	4	0
Weight evolution according to owner			
Gained weight	19 (23%)	41 (20%)	8 (53%)
Lost weight	8 (9.6%)	18 (9.0%)	1 (6.7%)
Stable	56 (67%)	142 (71%)	6 (40%)
Unknown	2	3	0
Perception with visual scale			
Agreement	9 (14%)	108 (72%)	7 (47%)
Overestimation	6 (9.2%)	20 (13%)	7 (47%)
Underestimation	50 (77%)	21 (14%)	1 (6.7%)
Unknown	20	55	0

Characteristics related to dogs’ owners and weight management

**Characteristic**	**Underestimation**, N = 85	**Agreement**, N = 204	**Overestimation**, N = 15
School			
ENVA	54 (64%)	127 (62%)	6 (40%)
ENVT	31 (36%)	77 (38%)	9 (60%)
Number of people in the house			
One	13 (15%)	49 (24%)	2 (13%)
> 1	72 (85%)	155 (76%)	13 (87%)
Children in the house [Yes]	29 (34%)	50 (25%)	5 (33%)
Time of unleashed activity per week			
< 1h	31 (38%)	56 (28%)	4 (29%)
1-4h	18 (22%)	48 (24%)	5 (36%)
4-6h	14 (17%)	35 (18%)	0 (0%)
> 6h	18 (22%)	61 (30%)	5 (36%)
Unknown	4	4	1
Time of leashed activity per week			
<1h	23 (28%)	64 (32%)	7 (50%)
1-4h	16 (19%)	59 (30%)	0 (0%)
4-6h	22 (27%)	36 (18%)	2 (14%)
>6h	22 (27%)	38 (19%)	5 (36%)
Unknown	2	7	1
Sport [Yes]	20 (24%)	38 (19%)	4 (27%)
Unknown	0	6	0
Evolution of activity since last year			
Decreased	17 (23%)	37 (21%)	5 (36%)
Increased	9 (12%)	22 (13%)	1 (7.1%)
Stable	49 (65%)	116 (66%)	8 (57%)
Unknown	10	29	1
Housing			
Flat	40 (47%)	109 (54%)	7 (47%)
House	45 (53%)	90 (45%)	8 (53%)
Other	0 (0%)	3 (1.5%)	0 (0%)
Unknown	0	2	0
Stairs [Yes]	41 (49%)	97 (49%)	7 (50%)
Unknown	2	8	1
Outdoor access [Yes]	63 (76%)	135 (67%)	11 (73%)
Unknown	2	1	0
Outdoor type			
Balcony	12 (18%)	31 (22%)	1 (10%)
Garden	48 (73%)	104 (74%)	9 (90%)
Kennel	6 (9.1%)	6 (4.3%)	0 (0%)
Unknown	19	63	5
Access to a garden [Yes]	47 (57%)	104 (51%)	9 (60%)
Unknown	2	1	0
Outdoor frequency			
Rarely	3 (3.5%)	5 (2.5%)	2 (14%)
Sometimes	14 (16%)	31 (16%)	3 (21%)
Once a day	21 (25%)	43 (22%)	0 (0%)
Several times a day	47 (55%)	121 (60%)	9 (64%)
Unknown	0	4	1
Outdoor time per day			
< 30min	7 (8.4%)	13 (6.7%)	0 (0%)

0.5-2h	56 (67%)	126 (65%)	10 (67%)
> 2h	20 (24%)	54 (28%)	5 (33%)
Unknown	2	11	0
Play frequency			
Rarely	3 (3.7%)	12 (6.2%)	1 (7.1%)
Sometimes	17 (21%)	33 (17%)	1 (7.1%)
Once a day	27 (33%)	62 (32%)	6 (43%)
Several times a day	34 (42%)	85 (44%)	6 (43%)
Unknown	4	12	1
Other pet [Yes]	38 (45%)	103 (50%)	8 (53%)
Other dog [Yes]	19 (22%)	77 (38%)	4 (27%)
Unknown	0	1	0
Other cat [Yes]	26 (31%)	47 (24%)	6 (40%)
Unknown	0	4	0
Toys			
No	2 (2.4%)	11 (5.5%)	1 (7.1%)
Yes but doesn't play	17 (20%)	42 (21%)	2 (14%)
Yes	64 (77%)	146 (73%)	11 (79%)
Unknown	2	5	1
Sleeping location			
Bedroom	31 (46%)	66 (43%)	4 (40%)
Garage	1 (1.5%)	4 (2.6%)	1 (10%)
Kitchen	6 (8.8%)	5 (3.2%)	0 (0%)
Living room	23 (34%)	62 (40%)	3 (30%)
Special place	7 (10%)	17 (11%)	2 (20%)
Unknown	17	50	5
Sleeping location bis			
Inside	80 (95%)	195 (97%)	13 (93%)
Outside	4 (4.8%)	7 (3.5%)	1 (7.1%)
Unknown	1	2	1
Sleeps in bed [Yes]	26 (31%)	51 (25%)	3 (20%)
Sleeps in sofa [Yes]	21 (25%)	52 (25%)	2 (13%)
Sleeps in a basket [Yes]	40 (47%)	109 (53%)	2 (13%)
Sleeps on a mat [Yes]	22 (26%)	60 (29%)	5 (33%)
Sleeps on the floor [Yes]	18 (21%)	50 (25%)	5 (33%)
Sleeps in a high location [Yes]	3 (3.5%)	10 (4.9%)	0 (0%)

Characteristics related to dogs' activity and environment

**Characteristic**	**Underestimation**, N = 85	**Agreement**, N = 204	**Overestimation**, N = 15
Bowl			
Anti-glutton	9 (11%)	37 (18%)	1 (7.1%)
Classic	74 (89%)	164 (81%)	13 (93%)
Dispenser	0 (0%)	1 (0.5%)	0 (0%)
Unknown	2	2	1
Food type			
Industrial	67 (80%)	168 (83%)	12 (86%)
Industrial + homemade	15 (18%)	29 (14%)	1 (7.1%)
Homemade ration	2 (2.4%)	6 (3.0%)	1 (7.1%)
Unknown	1	1	1
Diet moisture			
Dry	64 (85%)	175 (91%)	11 (92%)
Dry + Wet	8 (11%)	12 (6.2%)	0 (0%)
Wet	3 (4.0%)	6 (3.1%)	1 (8.3%)
Unknown	10	11	3
Food quantity			
Ad_libitum	15 (21%)	45 (24%)	2 (17%)
According to the packaging tag	35 (48%)	92 (48%)	5 (42%)
According to a vet	23 (32%)	54 (28%)	5 (42%)
Unknown	12	13	3
Feeding frequency			
Once a day	15 (21%)	43 (22%)	2 (17%)
Twice a day	43 (60%)	112 (58%)	9 (75%)
3-4 times per day	0 (0%)	7 (3.6%)	0 (0%)
Self distribution	14 (19%)	30 (16%)	1 (8.3%)
Unknown	13	12	3
Food store			
Supermarket	18 (25%)	38 (21%)	2 (17%)
Pet shop	45 (62%)	103 (57%)	9 (75%)
Vet	9 (12%)	40 (22%)	1 (8.3%)
Unknown	13	23	3
Eats leftovers [Yes]	43 (57%)	93 (50%)	5 (42%)
Unknown	9	17	3
Eats rewards [Yes]	56 (74%)	145 (76%)	11 (85%)
Unknown	9	14	2
Feeding location			
Inside	69 (90%)	175 (91%)	11 (85%)
Outside	8 (10%)	18 (9.3%)	2 (15%)
Unknown	8	11	2
Number of people serving the meals			
1	27 (34%)	71 (37%)	4 (31%)
2	33 (41%)	86 (44%)	6 (46%)
> 2	20 (25%)	37 (19%)	3 (23%)
Unknown	5	10	2
How food is quantified			
Not quantified	22 (28%)	45 (23%)	1 (7.7%)
Approximatively	3 (3%)	3 (2%)	1 (7.7%)
With a cup	47 (59%)	131 (66%)	10 (77%)
Wheighed	8 (10%)	18 (9%)	1 (7.7%)
Unknown	5	7	2

Begging behavior [Yes]	20 (26%)	68 (35%)	3 (23%)
Unknown	9	9	2
Regularly dewormed [Yes]	45 (55%)	118 (61%)	14 (93%)
Unknown	3	12	0
Treats (% of energy)	9 (3, 11)	5 (2, 9)	5 (1, 8)
Unknown	0	1	0

Characteristics related to dogs' diets and food management

CATS

**Characteristic**	**Underestimation**, N = 65	**Agreement**, N = 171	**Overestimation**, N = 34
Sex			
Female	34 (52%)	84 (49%)	21 (62%)
Male	31 (48%)	87 (51%)	13 (38%)
Age	3.5 (2.0, 6.0)	4.0 (2.0, 7.0)	4.8 (2.3, 10.5)
Age category			
Young adult	53 (82%)	128 (75%)	18 (53%)
Mature adult	6 (9.2%)	25 (15%)	4 (12%)
Senior	6 (9.2%)	18 (11%)	12 (35%)
Neutering			
No	2 (3%)	17 (10%)	1 (3%)
Yes	62 (97%)	154 (90%)	33 (97%)
Unknown	1	0	0
Breed			
Bengal	0 (0%)	2 (1.2%)	0 (0%)
Birman	1 (1.5%)	4 (2.3%)	0 (0%)
British Shorthair	0 (0%)	1 (0.6%)	0 (0%)
Chartreux	2 (3.1%)	0 (0%)	1 (2.9%)
Crossbreed	58 (89%)	154 (90%)	29 (85%)
Exotic Shorthair	0 (0%)	0 (0%)	1 (2.9%)
Highland Fold	0 (0%)	1 (0.6%)	0 (0%)
Maine Coon	0 (0%)	1 (0.6%)	1 (2.9%)
Norvegian	1 (1.5%)	1 (0.6%)	1 (2.9%)
Persian	0 (0%)	4 (2.3%)	0 (0%)
Ragdoll	1 (1.5%)	0 (0%)	0 (0%)
Siamese	2 (3.1%)	3 (1.8%)	1 (2.9%)
Purebreed [Yes]	7 (11%)	17 (9.9%)	5 (15%)
Hairs			
Short	43 (66%)	117 (69%)	24 (71%)
Medium	15 (23%)	38 (22%)	8 (24%)
Long	7 (11%)	15 (8.8%)	2 (5.9%)
Unknown	0	1	0
BCS by a veterinarian			
2	0 (0%)	0 (0%)	2 (5.9%)
3	0 (0%)	2 (1.2%)	1 (2.9%)
4	0 (0%)	5 (2.9%)	16 (47%)
5	12 (18%)	94 (55%)	10 (29%)
6	39 (60%)	40 (23%)	2 (5.9%)
7	8 (12%)	28 (16%)	3 (8.8%)
8	5 (7.7%)	1 (0.6%)	0 (0%)
9	1 (1.5%)	1 (0.6%)	0 (0%)
Status			
Underweight	0 (0%)	7 (4.1%)	19 (56%)
Ideal	12 (18%)	94 (55%)	10 (29%)
Overweight	53 (82%)	70 (41%)	5 (15%)

Cats' characteristics

**Characteristic**	**Underestimation**, N = 65	**Agreement**, N = 171	**Overestimation**, N = 34
Owner age			
< 40	47 (73%)	130 (77%)	19 (58%)
≥ 40	17 (27%)	39 (23%)	14 (42%)
Unknown	1	2	0
BCS by the owner			
1	0 (0%)	1 (0.7%)	1 (3.1%)
3	4 (7.3%)	10 (7.1%)	3 (9.4%)
4	4 (7.3%)	2 (1.4%)	4 (12%)
5	24 (44%)	66 (47%)	11 (34%)
6	5 (9.1%)	20 (14%)	5 (16%)
7	14 (25%)	39 (28%)	6 (19%)
8	1 (1.8%)	1 (0.7%)	1 (3.1%)
9	3 (5.5%)	1 (0.7%)	1 (3.1%)
Unknown	10	31	2
Owner’s preference			
Make the cat gain weight	5 (7.9%)	5 (3.0%)	3 (9.1%)
Make the cat maintain that weight	49 (78%)	104 (63%)	21 (64%)
Make the cat lose weight	9 (14%)	56 (34%)	9 (27%)
Unknown	2	6	1
Weighing numbers since last year			
Never	10 (16%)	26 (16%)	7 (21%)
Once	41 (65%)	95 (57%)	18 (53%)
Several times	12 (19%)	45 (27%)	9 (26%)
Unknown	2	5	0
Weight change since last year according to the owner			
Gained	13 (21%)	35 (21%)	8 (24%)
Lost	7 (11%)	12 (7.2%)	4 (12%)
Stable	43 (68%)	120 (72%)	22 (65%)
Unknown	2	4	0
Regularly dewormed [Yes]	21 (34%)	66 (40%)	10 (29%)
Unknown	3	7	0
Perception with visual scale			
Agreement	9 (16%)	92 (66%)	9 (28%)
Overestimation	11 (20%)	27 (19%)	21 (66%)
Underestimation	35 (64%)	21 (15%)	2 (6.2%)
Unknown	10	31	2

Characteristics related to cats’ owners and weight management

**Characteristic**	**Underestimation**, N = 65	**Agreement**, N = 171	**Overestimation**, N = 34
School			
ENVA	44 (68%)	106 (62%)	13 (38%)
ENVT	21 (32%)	65 (38%)	21 (62%)
More than one people in the housing [Yes]	52 (80%)	127 (74%)	30 (88%)
Children in the housing [Yes]	22 (34%)	43 (25%)	12 (35%)
Activity_score			
2	4 (6.5%)	4 (2.4%)	0 (0%)
3	3 (4.8%)	19 (11%)	3 (8.8%)
4	10 (16%)	14 (8.4%)	6 (18%)
5	12 (19%)	27 (16%)	5 (15%)
6	14 (23%)	41 (25%)	8 (24%)
7	9 (15%)	24 (14%)	5 (15%)
8	5 (8.1%)	25 (15%)	5 (15%)
9	3 (4.8%)	7 (4.2%)	1 (2.9%)
10	2 (3.2%)	5 (3.0%)	1 (2.9%)
Unknown	3	5	0
Dwelling			
Flat	41 (64%)	111 (66%)	21 (62%)
House	23 (36%)	55 (33%)	13 (38%)
Other	0 (0%)	1 (0.6%)	0 (0%)
Unknown	1	4	0
Lifestyle			
Indoor only	23 (35%)	61 (36%)	14 (41%)
Outdoor access	42 (65%)	110 (64%)	20 (59%)
Time spent in a high place			
Never	2 (3.2%)	3 (1.8%)	2 (6.2%)
Sometimes	10 (16%)	32 (20%)	4 (12%)
Often	50 (81%)	129 (79%)	26 (81%)
Unknown	3	7	2
Access to a high place [Yes]	49 (77%)	134 (80%)	25 (76%)
Unknown	1	4	1
Other animals [Yes]	29 (46%)	74 (45%)	15 (44%)
Unknown	2	6	0
Dog [Yes]	10 (16%)	23 (14%)	7 (21%)
Unknown	2	6	0
Other cat [Yes]	23 (37%)	59 (36%)	15 (44%)
Unknown	2	6	0
Toys [Yes]	40 (63%)	100 (61%)	21 (62%)
Unknown	2	6	0

Characteristics related to cats' activity and environment

**Characteristic**	**Underestimation**, N = 65	**Agreement**, N = 171	**Overestimation**, N = 34
Food type			
Canned	1 (1.6%)	8 (4.8%)	0 (0%)
Dry	37 (60%)	95 (57%)	24 (73%)
Dry and wet	24 (39%)	63 (38%)	9 (27%)
Unknown	3	5	1
Number of food distribution(s) per day			
1	7 (11%)	18 (11%)	5 (15%)
2	5 (7.9%)	18 (11%)	4 (12%)
3-4	9 (14%)	34 (21%)	4 (12%)
> 4	2 (3.2%)	3 (1.9%)	0 (0%)
Ad_libitum	40 (63%)	89 (55%)	20 (61%)
Unknown	2	9	1
Food store			
Supermarket	30 (49%)	72 (44%)	12 (36%)
Pet shop	25 (41%)	66 (40%)	19 (58%)
Vet	6 (9.8%)	27 (16%)	2 (6.1%)
Unknown	4	6	1
Grain free diet [Yes]	3 (4.8%)	4 (2.4%)	4 (12%)
Unknown	3	4	1
“Light” diet [Yes]	51 (82%)	127 (76%)	26 (79%)
Unknown	3	4	1
Vegan diet [Yes]	3 (4.8%)	8 (4.8%)	0 (0%)
Unknown	3	4	1
How food is quantified			
Never	37 (64%)	116 (71%)	25 (78%)
With a cup	18 (31%)	37 (23%)	6 (19%)
Weighing	3 (5.2%)	10 (6.1%)	1 (3.1%)
Unknown	7	8	2
Dry food frequency			
Never	2 (3.1%)	4 (2.3%)	0 (0%)
Often	0 (0%)	1 (0.6%)	0 (0%)
Everyday	63 (97%)	166 (97%)	34 (100%)
Wet food frequency			
Never	20 (31%)	60 (35%)	18 (53%)
Sometimes	10 (15%)	10 (5.8%)	3 (8.8%)
Often	35 (54%)	101 (59%)	13 (38%)
Meat and fish frequency			
Never	38 (58%)	107 (63%)	20 (59%)
Sometimes	18 (28%)	44 (26%)	3 (8.8%)
Often	9 (14%)	20 (12%)	11 (32%)
Treats frequency			
Never	38 (58%)	97 (57%)	26 (76%)
Sometimes	17 (26%)	35 (20%)	4 (12%)
Often	10 (15%)	39 (23%)	4 (12%)
Begging behavior [Yes]	13 (21%)	44 (26%)	5 (15%)
Unknown	2	3	0
Leftovers frequency			
Never	23 (35%)	58 (34%)	16 (47%)
Sometimes	19 (29%)	52 (30%)	2 (5.9%)
Often	23 (35%)	61 (36%)	16 (47%)

Characteristics related to cats' diets and food management
