# Supplementary material for: The Perception of the Body Condition of Cats and Dogs by French Pet Owners and the Factors Influencing Underestimation
Source: Animals (Basel). 2023 Nov 25;13(23):3646. doi: 10.3390/ani13233646 (PMC10705725; doi:10.3390/ani13233646)
Supplement: Supplementary file 1 [file animals-13-03646-s001.zip › Supplementary Tables.pdf]

BCS by a veterinarian / Owners' opinion	3	4	5	6	7	8	9
**Skinny**	3	5	4	0	0	0	0
**Optimal**	2	17	151	56	10	1	0
**A bit fat**	0	0	14	19	15	4	2
**Very fat**	0	0	0	0	1	0	0

Table S1: Dog owners' opinion according to dogs' BCS rated by a veterinarian.

In green the agreement, in blue overestimation by the owner and in red underestimation by the owner.

BCS by a veterinarian / Owners' opinion	2	3	4	5	6	7	8	9
**Skinny**	1	2	5	12	1	0	0	0
**Optimal**	1	1	15	94	38	8	0	0
**A bit fat**	0	0	1	10	40	28	5	1
**Very fat**	0	0	0	0	2	3	1	1

Table S2: Cat owners' opinion according to cats' BCS rated by a veterinarian.

In green the agreement, in blue overestimation by the owner and in red underestimation by the owner.

BCS by a veterinarian / BCS by the owner	3	4	5	6	7	8	9
1	1	1	0	0	0	0	0
2	0	0	1	0	0	0	0
3	2	6	7	2	0	0	0
4	1	4	9	1	0	0	0
5	1	5	97	36	4	0	0
6	0	0	1	10	1	0	0
7	0	0	10	12	10	2	2
8	0	0	0	0	1	1	0
9	0	0	0	0	1	1	0

Table S3: Dogs' BCS rated by owners with the visual scale according to BCS rated by a veterinarian.

In green the agreement, in blue overestimation by the owner and in red underestimation by the owner.

BCS by a veterinarian / BCS by the owner	2	3	4	5	6	7	8	9
1	0	0	2	0	0	0	0	0
2	0	0	0	0	0	0	0	0
3	1	2	4	9	1	0	0	0
4	0	0	4	6	0	0	0	0
5	1	1	7	65	24	3	0	0
6	0	0	1	7	17	5	0	0
7	0	0	1	4	29	21	4	0
8	0	0	0	0	0	3	0	0
9	0	0	0	0	1	2	1	1

Table S4: Cats' BCS rated by owners with the visual scale according to BCS rated by a veterinarian.

In green the agreement, in blue overestimation by the owner and in red underestimation by the owner.

	Dogs			Cats		
	Lose	Maintain	Gain	Lose	Maintain	Gain
**Underweight**	0	2	3	1	17	7
**Ideal**	9	163	13	5	101	5
**Overweight excluding obese**	28	70	1	62	54	1
**Obese**	5	2	0	6	1	0

Table S5: Owners' will about their pets' weight.

In blue: attitude leading to underweight
In red: attitude leading to overweight

**Variable**	**Agreement**, N = 204	**Underestimation**, N = 85	**p-value**
Multiple people in the household [Yes]	155 (76%)	72 (85%)	0.10
Children [Yes]	50 (25%)	29 (34%)	0.095
Owner age > 40 yo	58 (29%)	30 (36%)	0.2
Sex [Male]	111 (54%)	36 (42%)	0.062
Time of leashed activity per week > 4h	74 (38%)	44 (53%)	0.017
Outdoor access	135 (67%)	63 (76%)	0.12
Other dog in the household	77 (38%)	19 (22%)	0.011
Cat in the household	47 (24%)	26 (31%)	0.2
Anti-glutton bowl	37 (18%)	9 (11%)	0.12
Food store			0.2
Supermarket	38 (21%)	18 (25%)	
Pet shop	103 (57%)	45 (62%)	
Vet	40 (22%)	9 (12%)	
Gready [Yes]	68 (35%)	20 (26%)	0.2
Treats (% of daily energy intake)	5 (2 - 9)	9 (3 – 11)	0.006
Status [Overweight]	34 (17%)	73 (86%)	<0.001
Dog's age	4 (2 – 8)	5 (2 – 9)	0.2

Table S6: Results of the univariate analysis for dog owners' underestimation
Only variables with p<0.25 shown

**Variable**	**Agreement**, N = 171	**Underestimation**, N = 65	**p-value**
Children [Yes]	43 (25%)	22 (34%)	0.2
Neutering [Yes]	154 (90%)	62 (97%)	0.088
Wet food			0.063
Never	60 (35%)	20 (31%)	
Sometimes	10 (6%)	10 (15%)	
Often	101 (59%)	35 (54%)	
Food ad libitum	89 (52%)	40 (62%)	0.2
Status [Overweight]	70 (41%)	53 (82%)	<0.001

Table S7: Results of the univariate analysis for cat owners' underestimation
Only variables with p<0.25 shown
